# Valorisation of agricultural biomass-ash with CO_2_

**DOI:** 10.1038/s41598-020-70504-1

**Published:** 2020-08-14

**Authors:** Colin D. Hills, Nimisha Tripathi, Raj S. Singh, Paula J. Carey, Florence Lowry

**Affiliations:** 1 Indo:UK Centre for Environment Research and Innovation, University of Greenwich, Chatham Maritime, Kent, UK; 2 Indo:UK Centre for Environment Research and Innovation, CSIR-Central Institute of Mining and Fuel Research, Dhanbad, India

**Keywords:** Environmental sciences, Engineering, Materials science

## Abstract

This work is part of a study of different types of plant-based biomass to elucidate their capacity for valorisation via a managed carbonation step involving gaseous carbon dioxide (CO_2_). The perspectives for broader biomass waste valorisation was reviewed, followed by a proposed closed-loop process for the valorisation of wood in earlier works. The present work newly focusses on combining agricultural biomass with mineralised CO_2_. Here, the reactivity of selected agricultural biomass ashes with CO_2_ and their ability to be bound by mineralised carbonate in a hardened product is examined. Three categories of agricultural biomass residues, including shell, fibre and soft peel, were incinerated at 900 ± 25 °C. The biomass ashes were moistened (10% w/w) and moulded into cylindrical samples and exposed to 100% CO_2_ gas at 50% RH for 24 h, during which they cemented into hardened monolithic products. The calcia in ashes formed a negative relationship with ash yield and the microstructure of the carbonate-cementing phase was distinct and related to the particular biomass feedstock. This work shows that in common with woody biomass residues, carbonated agricultural biomass ash-based monoliths have potential as novel low-carbon construction products.

## Introduction

This paper discusses environmental issues that are of relevance to the sustainable use of resources and protection of the environment. By re-using abundantly available agricultural biomass residues, and by reducing the amount of cement used in construction and their associated carbon emissions, significant potential sustainability gains can be realised. In consideration of this, a low carbon management option for biomass residues is proposed.

Agricultural activities generate huge volumes of biomass residues, with crop derived waste accounting for 94% of global biomass production^1^. These include cereals (wheat, rice, maize, and barley), sugarcane, soybean, and some oil crops, fruits, vegetables, roots and tubers, and sugar beet^1^. These residues are expected to increase as the world’s population exceeds 11 Billion by 2,100^2^.

It is estimated that globally 140 Gt of agricultural residues are generated each year^3,4^. According to Lal (2019)^5^, the total annual amount of crop residue produced globally is estimated at 2.8 Gt for cereal crops, 3.1 Gt for 17 major cereals and legumes, and 3.8 Gt for 27 common food crops. The IEA CCC (2015)^6^ reports the global resource for unexploited cereal crop residues amounts to 517 Mt.

The FAO (2002) ^7^ projects that from 1999 to 2030, the agricultural sector of developing countries will increase by 13% or 120 M ha. Predictions for global cereal yield suggest an increase in the range 0.9% per annum over the period 2005/2007–2050, continuing a trend of long-term declining yield growth (20 y average yield increase declined from ~ 3% pa in 1982, to ~ 1% by 2005)^8,9^. The intensification of agricultural and increased crop yield (producing more per unit of land) will undoubtedly raise crop residue production^10^.

A positive correlation exists between crop-residue availability and its production (e.g. the ratio of the amount of straw to grain, as in the case of cereals), based on the residue-to-crop ratio in a given country, region or at global scale^11–14^. Geographical location may also influence the crop residue to production relationship^15^. Some studies assume a directly proportional relationship between the amount of crop residue and crop yield (production per area unit), rather than total crop production^16,17^. The recoverable fraction (i.e. crop yield) could be as much as 25% of harvested residue and 90% as the processing residue^18,19^.

Agricultural biomass wastes and residues from primary production are found in the form of crops stalks, leaves, roots, fruit peels, seeds and nut shells. These wastes are not consumed directly as food or other by-products and their managed disposal is very important. Globally, 66% of the residual plant biomass comes from cereal straw (stem, leaf, and sheath material), with over 60% residues produced in low-income countries^20^. The major crops, including wheat, maize, rice, soybean, barley, rape seed, sugar cane, sugar beet have an annual global residue potential of about 5Gt^17^. Table 1 shows the production of fruit and vegetable and other residues; the former two being particularly in increasing demand, as the global population grows.

**Table 1:** Global production of fruits, vegetables and residues.

	Production (Mt)	References
**Fruits**		
Citrus	124.7	^83^
Banana	114.1	
Apple	84.6	
Grapes	74.5	
Mango, Mangosteen and Guava	45.2	
Pineapple	25.4	
**Vegetables**		
Potato	388	
Tomato	171	
Cabbage and other Brassicas	71.8	
Carrot and Turnip	38.8	
Cauliflower and Broccoli	24.2	
Peas	17.4	
**Residues (from peeling and processing of fruit and vegetables) (% w/w, total weight)**		^84–86^
Citrus fruit	50	
Banana	35	
Grapes	20	
Potato	15	
Jackfruit	50–70	

Traditionally, root and tuber crops are the mainstay of food in many countries, providing 45% of the world's carbohydrate supply, as well as for animal feedstocks and industrial products (e.g., starch, distilled spirits etc.). The projected annual global demand for root and tuber crops per capita is expected to increase from 69 to 75 kg/y from 1999 to 2050. The major root and tuber crops are potato, sweet potato, cassava and yam are key to maintaining food security and the promotion of better livelihoods through value chain opportunities^21^. Cassava, for example, has potential for biofuel production, which is projected to increase from the 1 Mt/y in 2005, to around 8 Mt/y by 2050. In China, the use of cassava as a feedstock for biofuel production is projected to increase from 0.4% in 2005 to 1.8% by 2050^22,23^, suggesting that demand could be even higher. The tentative forecast for world cassava production for 2015 was 289 Mt, 0.5 Mt more than 2014. However, in sub-Saharan Africa production in 2015 was 163 Mt, showing a decline of 3 Mt from 2014, primarily resulting from adverse weather conditions^24^.

Nuts and drupes (fleshy thin-skinned fruit with a central stone and seed) are another important food source. Globally, 72% of total global drupe production involved coconut and mango, and the remaining 28% from nuts (almonds, pistachio and walnuts), stone fruit (cherries, peach, plum and nectarines) and olives, originating in Poland, Turkey, Japan, Spain, Italy and Greece^25,26^. The global annual availability of drupe endocarp biomass (residue) is primarily driven by coconut production and ranges from 24 to 31 Mt/y^26,27^. The other substantial residue is generated from the cashew apples (i.e. the juicy swollen pedicel). About 95% of the global cashew apple crop is allowed to rot^28^, and in India 98% (3.9 Mt/y) is lost this way^29^.

As indicated above, the global use of hydraulic cement, which is consumed in bulk, is second only to water. In 2018, consumption reached 4.1 Gt^30^, and is projected to rise to 4.8 Gt/y by 2030^31^. China is the largest cement producing country (about 60% of global production) followed by India (7%). To mitigate the impact of cement production, associated emissions require management by, for example, carbon capture, utilization and storage (CCUS) technologies and the use of lower-temperature (lower carbon intensive) alternative clinkers or both. As discussed in the present work, the use of agricultural wastes may have a role to play.

### Challenges/issues from an environmental perspective

Biomass and their residues are low-cost voluminous material resources that are generally environmentally benign, often being returned to the soil as an enriching media. Greater than 2 Gt of unused crop residues are dumped in municipal landfills or burned by households in developing countries^32^. These activities contribute to 18% of total global CO_2_ emissions^33–36^.

The use of biomass waste to produce energy and other products is of mounting interest. In this regard, the energy potential of residues, in 2050, has been estimated to be in a range of 15–280 EJ yr^−1^ globally^37–40^. However, this estimation of biomass residues generated or their potential for alternative uses is approximate^41^. As such, the global availability of crop residues has been re-examined by Tripathi et al. (2019)^36^.

In most developed and developing countries, the collection, recycling and sustainable disposal of the increasing quantities of biomass and other solid wastes are the major challenges. Their conversion into energy and other products can reduce environmental harms and generate much needed value, particularly in developing countries with large quantities of available biomass.

By far, the greatest use of biomass wastes has been as an energy source by direct combustion of wood and crop residues^42^ and in developing countries, biomass is dominantly used as a fuel in open fires for cooking and heating^43,44^. Globally, nearly three billion people rely on biomass-based fuels sourced from wood or charcoal for cooking and heating^45^. However, for biomass-based power generation, the EU and USA account for most^46^. Biomass burned in Europe to produce energy is projected to contribute 20% of the European renewable energy target by 2020^47^. As biomass incineration and pyrolysis generates substantial amounts of ash and CO_2_, innovative management strategies that can incorporate both are timely. Ideally, biomass ash should be returned to land as an enrichment, but energy from waste generates large volumes of ash that fall within waste management regulations, necessitating management by for example, landfilling. These landfill ‘deposits’ are a relatively consistent potential resource for manufactured products.

Globally, the demand for ‘carbon efficient’ management solutions to minimise CO_2_ emissions and utilise waste is increasing^48^. The Paris agreement recommendations are for immediate action to keep the global increase in temperatures below 1.5 °C^49^. As carbon capture and storage (CCS) in the geosphere is slow to mature, there is a need to explore carbon capture and utilisation (CCU) for the management of point source CO_2_ emissions. Indeed, emerging CCU technologies offer significant opportunities for the management of biomass waste coupled with value-addition and CO_2_ emissions reductions (hence the acronym, CCUS).

As a recent example, in November 2019, in Delhi and its adjacent major state, Punjab, suffered record levels of smog and poor air quality. The major contributor (about 50%) was stubble burning by the farmers. In one single day 5,953 fires burned and a monthly total of 31,267 fires was recorded^50^.

As mentioned, the production of cement has a high associated carbon footprint, as calcination generates large amount of CO_2_ gas (approximately 650–750 kg CO_2_/t of cement produced). Some 7% of worldwide greenhouse gas emissions are attributed to cement production^51^.

During 2014–2017, the IEA (2019)^30^ reported an annual increase of 0.5% in clinker-to-cement ratio, resulting into an increase of 0.3%/y in the direct CO_2_ intensity of cement production. This report emphasised the need for an annual decline in emissions of 0.7% by 2030 and deployment of CCUS-based technologies to achieve a sustainable emissions reduction scenario.

An approach that can combine biomass waste management with a reduction of cement production-related emissions is described below. Thus, the present work involves the transformation of CO_2_ into mineral carbonates on biomass ash, in a way previously described for other thermal or mineral wastes^52,53^.

## Materials and methods

In the present study, biomass residues are categorised as: shell, fiber and soft peel wastes. The biomass residues derived from shell include cobnut, coconut, walnut, almond and peanut; fiber includes jute (hemp), flax, barley straw, hay, and husks from rice and sugarcane; and soft peel includes sweet lime, orange, banana, yam, cassava, potato and pomegranate. The biomass residues described were sourced from India, Africa and the UK.

The residues were ashed in a muffle furnace at 900 ± 25 °C, over 4 h and then examined for (1) selected physical properties (e.g. particle size, bulk density, surface area and ash content) and (2) chemical composition (total carbon, elemental and phase-chemistry).

The particle size distribution of ashes was measured by laser diffraction analysis (Malvern Mastersizer MS2000) and bulk density by loose compaction in cylindrical holders (expressed as kg/m^3^). The surface area was determined (Micromeritics Gemini V2.00), and total carbon was analysed by CHN analysis (FLASH EA 1112 Series). The bulk elemental composition was determined by X-ray fluorescence spectrometry (Philips LW1400 and XRFWIN software).

The biomass ashes were moistened (20% w/w, total weight) to examine their reactivity to pure CO_2_ at a pressure of ~ 2 bar. The ashes were exposed to CO_2_ for four-separate cycles in a closed pressurised carbonation chamber, with the first three cycles extending to one hour each, and the fourth cycle being 24 h. The uptake of CO_2_ in ashes was determined on weight gain (% w/w, total weight) basis. This approach was taken as the results obtained correlate closely to those experienced during the carbonation of wastes in commercial facilities^36,54^.

### Product development and characterisation

For the production of monolithic specimens, biomass ashes were moistened (10% w/w, total weight) using a dropper followed by thorough hand-mixing, before being cast as small monolithic cylinders (7 mm × 7 mm—a similar size to manufactured carbonated aggregates). The casting process involved placing the moist ash in the mould followed by hand tamping and the top surface being struck, using a straight-sided spatula. Cylinders in their moulds were placed in a closed curing chamber containing pure CO_2_ at 50% RH. After 1 h, samples were de-moulded, and returned to the CO_2_ chamber to complete their cycle of 24 h exposure. Non-carbonated samples were treated similarly, but without exposure to CO_2_ and were regularly too fragile to demould after curing in air had been completed.

Some of the biomass ashes (including wood biomass ashes) are discussed in Tripathi and Hills et al. 2020^54^. As mentioned earlier, it was necessary to add Portland cement raw biomass waste to provide a reference point, as Portland cement is a commonly used hydraulic cementitious binder. Raw biomass with and without Portland cement was mixed with fine sand (used as an inert mineral filler to change particle size distribution) and then cast into larger monolithic cylinders (3.4 cm × 3.4 cm) (Table 6). It should be emphasised that the Portland cement was used here for its ability to react with CO_2_ gas and produce calcium carbonate rather than its normal use as a hydraulic medium. Cylinders were cured in pure CO_2_ for one week.

### Assessment of CO_2_ uptake and strength in valorised biomass products

The CO_2_ uptake by the monoliths was calculated on weight gain (% w/w, total weight) basis and also by CHN analysis. The strength of these monolithic products was evaluated by applying a force until the cylinders failed. The strength was calculated by using the Eq. (1):

1 $${\sigma }_{c}=\frac{2.8 {F}_{c}}{\uppi {\mathrm{dm}}^{2}}$$

where $\sigma_{\text{c}}$ is the compressive strength in megapascals, F_*c* is_ the fracture load in kilonewtons, A*m* is the mean area of the cylinder, and d*m* is the mean diameter of the cylinder.

For each batch of carbonated cylinders, the average strength was calculated from the load recorded at failure, with the three axes of each cylinder being measured using digital callipers (Mecmesin MFG250).

The water resistance of carbonated ‘ash only’ monoliths was monitored by immersing them in tap water for 30 days to investigate their water sensitivity.

The biomass ashes and resultant carbonate-cemented products were investigated by X-Ray diffractometry and electron microscopy.

The biomass ashes without and with CO_2_ exposure were analysed with a Siemens D500 diffractometer, fitted with a Siemens K710 generator using 40 kV voltage and 40 mA current, between 5° and 65° 2θ. The interpretation of diffractograms was aided by DIFFRAC^*plus*^ EVA software (Bruker AXS) and Rietveld refinement.

The capture of back-scattered electron micrographs augmented by EDAX analysis (JEOL JSM-5310LV, Oxford Instruments Energy Dispersive Spectrometer-EDAX) was performed on polished resin blocks, for both carbonated and reference (no-carbonated) biomass-ash products.

## Results and discussion

By combining biomass residues (both raw and ashed) and CO_2_ gas into solid monolithic products a potential future ‘zero waste’ option for these residues is established. Indeed, 4 individual biomass wastes presented in this work have been recently examined by the authors^54^ and are used here as reference residues.

The potential of biomass waste with the *right* chemical and mineralogical composition to react with gaseous CO_2_ is harnessed to develop products that are analogous to those made with hydraulic cement. The manufactured products include those hardened by ‘ash only’ and where ash was used as a partial replacement for cement. Both approaches were used to encapsulate raw biomass in different combinations.

The potential for further innovation including the integration of a direct flue-gas capture and mineralisation step could have significant environmental benefits, not least as this ‘circular economic’ approach will reduce both the landfilling of biomass waste/residue and gaseous emissions, whilst protecting virgin resources. An offsetting of CO_2_ via the replacement of hydraulic cement will be a further added benefit. This ‘offset’ is of particular importance as we have seen, the cement industry is growing at the rate of about 2.5% pa generating 39.3 ± 2.4 Gt during the period 1928–2016, with 90% of this evolved since 1990^55^.

### Physical, chemical and mineralogical characteristics of biomass residues

The physical and chemical characteristics of the biomass residues as received, and their ashes are given in Table 2. The surface area of raw fibres was higher compared to shell and soft peel with the later having the lowest values recorded. The ashes from soft peel wastes have a higher surface porosity than most of the ash generated from fibre-waste. The particle size distribution of ashes was specific to the individual biomass feedstock (Table 2). The size range of ash particle size was 3–39 µm for soft peel, 14–52 µm for shell and 1.21–45 µm for fibre-derived ashes.

**Table 2:** Particle size and BET surface area of raw biomass and their ash.

Type of biomass	Biomass ash	Ash content (%)	Ash particle size D_50_ (µm)	Total carbon in ash (g/kg)	Surface area (m^2^/g)	
					Raw biomass (m^2^/g)	Biomass ash (m^2^/g)
Shell	Cobnut shell	0.70	18.14	8.54	0.93	2.61
	Coconut shell	0.31	–	5.18	1.99	5.87
	Walnut shell	0.30	52.3	12.41	0.63	1.77
	Almond shell	0.94	24.10	8.58	0.36	1.43
	Peanut shell	1.38	13.87	9.62	1.43	3.13
Fibre	Jute (hemp)	0.96	45.38	5.09	1.42	2.71
	Flax	1.41	28.33	7.45	1.52	3.94
	Straw (barley)	3.17	11.22	5.74	1.35	2.62
	Hay	1.98	16.22	6.25	1.14	3.50
	Rice husk	5.08	1.21	10.43	1.76	2.79
	Coconut husk (coir)	0.80	6.86	5.18	1.48	1.97
	Sugarcane husk	4.64	9.91	5.78	1.41	1.59
Soft peel	Sweet lime	3.06	43.20	17.28	0.93	2.99
	Banana	3.80	2.90	8.70	0.33	2.32
	Yam	6.36	33.5	10.86	0.72	0.85
	Cassava	5.23	37.0	5.31	0.88	1.67
	Potato	4.22	3.40	7.77	0.31	5.47
	Pomegranate	1.45	15.59	10.07	0.28	1.92
	Orange	3.10	38.83	7.92	0.64	1.25

The total carbon content of ashes varied with some soft peel waste giving a higher yield. A similar trend was observed for shell ash. The carbon content in all the fibre-derived ashes, except rice husk, was generally less variable and within in the same range (Table 2).

The oxide equivalent composition of ashes is given in Table 3. Walnut shell, jute (hemp) and sweet lime ashes had the highest CaO content in their respective biomass categories. Calcia is the key mineral indicator of the potential CO_2_ reactivity of these ashes. It should be noted that coconut shell ash, which had low ash content (0.3% w/w, total weight) was partially characterised as a potential candidate ash, but due to the lack of sample could not be fully evaluated in this study.

**Table 3:** Mineralogical composition of agricultural biomass ashes (% w/w, total weight).

Type of biomass	Biomass Ash	K_2_O	CaO	SO_3_	MgO	SiO_2_	P_2_O_5_	Al_2_O_3_	Na_2_O	Fe_2_O_3_	Cl	SrO	MnO	TiO_2_	ZnO
Shell	Cobnut shell	21.2	18.1	1.1	3.6	8.8	2.8	1.1	0.4	1.5	0.1	0.04	0.4	0.1	0.03
	Walnut shell	4.1	52.3	1.1	5.1	4.4	2.5	1.0	4.3	2.2	0.9	–	0.2	0.06	0.04
	Almond shell	24.4	24.1	14.4	3.4	4.2	3.8	1.1	0.9	0.7	–	0.5	0.08	0.04	0.02
	Peanut shell	21.8	13.9	12.4	11.7	7.8	3.1	2.4	1.8	1.6	0.6	0.3	0.2	0.1	0.04
Fibre	Jute (hemp)	10.2	45.4	2.5	2.6	5.2	2.1	0.7	0.2	1.2	–	0.09	0.09	–	0.04
	Flax	8.4	28.3	3.6	3.0	8.4	3.2	2.4	0.3	2.1	–	0.05	0.2	0.3	0.05
	Straw (barley)	23.7	11.2	3.1	1.3	26.0	1.8	1.1	1.1	0.4	0.4	0.03	0.1	–	0.03
	Hay	13.4	16.2	4.5	2.1	21.6	5.5	0.5	1.3	0.4	0.08	0.04	0.4	0.04	0.06
	Rice husk	3.6	1.2	0.08	2.5	68.2	6.7	0.4	0.1	0.4	–	0.05	0.1	0.03	0.02
	Coconut husk (coir)	14.5	6.9	3.6	1.0	18.1	1.9	1.8	2.0	2.1	17.0	0.1	0.05	0.2	0.03
	Sugarcane husk	9.2	9.9	5.1	4.3	25.6	3.4	3.6	0.8	2.7	0.08	0.03	0.06	0.3	0.03
Soft peel	Sweet lime	15.9	43.2	2.8	3.1	2.5	2.4	0.4	0.6	0.4	–	0.03	0.04	–	0.02
	Banana	36.5	2.9	1.2	0.7	7.2	2.7	0.4	0.1	0.3	1.9	0.06	0.07	–	0.02
	Yam	51.8	5.6	4.1	5.4	10.1	20.9	0	0.07	1.1	0	0.04	0.2	0.3	0.1
	Potato	27.8	3.4	2.2	0.07	4.1	3.6	0.6	0.4	0.3	0.2	0.09	0.02	–	0.04
	Cassava peel	15.2	17.6	5.4	5.9	35.2	5.7	9.8	0.2	2.5	0.07	0.1	0.8	0.9	0.2
	Pomegranate	23.6	15.6	6.3	3.7	1.6	1.4	0.5	0.5	0.4	0.2	0.2	0.04	0.04	0.03
	Orange	21.8	38.8	3.9	3.7	3.1	4.5	0.8	1.1	0.3	–	0.2	0.04	–	0.03
	Cassava pulp	6.4	37.8	3.3	16.3	4.4	2.7	0.3	0.8	0.5	–	0.2	0.5	–	0.1

Note: Over 10% is good for reasonable uptake.

Once manufactured, the carbonated ‘ash only’ monolithic specimens remained intact after immersion in water for 30 days, with no signs of physical degradation, except for the banana peel ash where some swelling and micro-cracking was observed; this was attributed to incomplete carbonation and the presence of CaO, which hydrated forming portlandite (detected by XRD) led to moisture-induced expansion.

### Ash content and CO_2_ reactivity

The major and minor elements including Al, Ca, Cl, Fe, O, K, Mg, P, Na, S, Mn, Si and Ti in biomass are the major ash forming elements. These elements are generally found in decreasing order of abundance^56^:

$${\text{O}} > {\text{Ca}} > {\text{K}} > {\text{Si}} > {\text{Mg}} > {\text{Al}} > {\text{Cl}} > {\text{P}} > {\text{Fe}} > {\text{Na}} > {\text{S}} > {\text{Mn}} > {\text{Ti}}$$

Vassilev et al. (2017)^56^ reported a negative relationship between calcia and ash content in individual species of wood, but not agricultural biomass where a positive relationship was identified. These observations are not universally applicable to woody biomass examined in our as yet unpublished work (where some positive relationships were found), and for the agricultural biomass examined here (where some negative relationships were observed). Some of the soft peel wastes had both high calcia and ash contents (Tables 2 and 3), indicating biomass waste is complex and heterogeneous in nature. By way of example, citrus fruit peels, including orange and sweet lime, have a ‘high’ calcia content; an observation also reported by Sweitzer (2018)^57^. The ash yield from citrus peel is higher than other ‘low’ calcia containing fruit peel -an observation also reported by Vassilev et al. (2017)^56^.

The fibres, jute (hemp) and flax, had low ash and high calcia content. This relationship is contrary to Vassilev et al. (2017)^56^ observations and may be attributed to these bast fibres having pectin and calcium ions ‘gluing’ constituent fibres together^58^. On the other hand, rice and sugarcane husk presented high ash and low calcia contents. Vassilev et al. (2017)^56^ reported extremely low CaO contents in rice husk, due to high ash yield; data that supports the negative CaO:ash yield-relationship discussed above.

Some shell-derived ashes were calcia rich, up to 52% w/w (total weight), with walnut as an example (Table 3). However, the ash content of shell waste was low, following the negative relationship previously described^56^. That said, the enrichment pattern is opposite to these previously reported findings that reported calcia depleted ash upon burning, which was not observed in our study.

The relationship observed in our work between ash yield and calcia content can be explained by the observation that a high ash results from combustion of biomass particularly enriched in silica/silicates. The ashes abundant in Ca, Cl, Fe, K, Mg, Na, P, S, chloride, sulphate, carbonate and phosphate tend to be of lower yield -also noted by Vassilev et al. (2013, 2014)^59,60^.

The ash arising from herbaceous biomass has been shown to vary with the part of the plant being combusted^61,62^; with grains having a lower ash content than their straw^63^. Other factors related to the ash content of agricultural biomass include:

1. the loss of nutrients from plants, as a wash-effect by precipitation or delayed harvest^64^,
2. the type of soil used for growing plants^65^, and
3. the season they are grown in, especially for grass species^61^.

As observed in the present study, some fruit peel, shell and fibre-biomass yielded higher calcia containing ashes (see Table 3); the key mineral indicator of CO_2_ reactivity.

## CO_2_ uptake in biomass ash and their products

### Biomass ashes

As mentioned, CO_2_-reactive biomass ashes represent a potential resource for the manufacture of value-added products. However, in an industrialised setting involving, e.g., energy from biomass waste, ashes will be classed as waste and, therefore, to meet ‘end of waste’ regulations (and to be legally declared as a product), carbonated ashes must: (1) be ‘fit for purpose’ by conforming to an agreed specification, (2) be risk managed, and (3) meet a market need.

**Figure 1:**
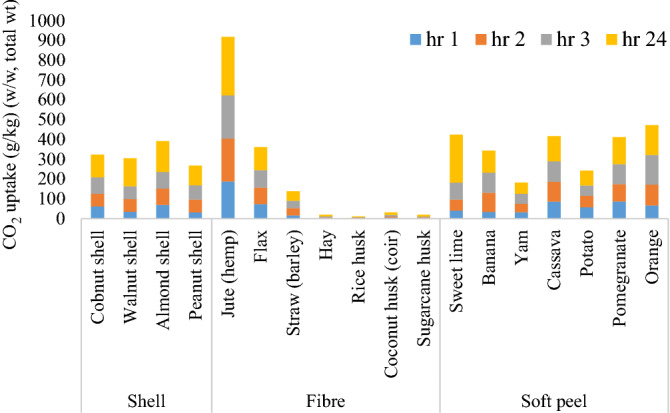
Cumulative CO_2_ uptake (w/w, total weight) in agricultural biomass ashes after 4 carbonation cycles.

After each successive cycle of exposure to carbon dioxide gas, a gradual increase in the amount of CO_2_ uptake was observed (Fig. 1, Table S1).

**Figure 2:**
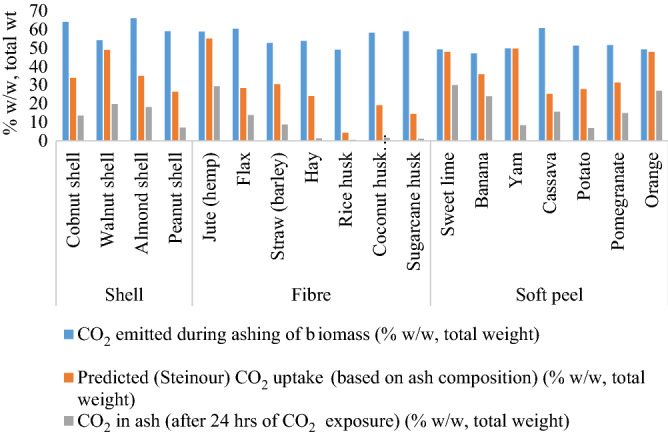
Theoretical and experimental CO_2_ uptake in biomass ashes.

The potential of ashes to mineralise CO_2_ was calculated theoretically, using *the* Steinour equation^66^. This equation uses the stoichiometry of an ash (taken from its oxide composition) to predict the maximum possible carbon ‘uptake’ as a % w/w (total weight). However, it should be noted that this equation and modifications thereof are not appropriate to all potentially carbonate-able wastes and the predictions are normally much more than can be achieved under laboratory/real-world conditions. The theoretical CO_2_-uptakes in w/w (total weight) in shell, fibre and soft peel ashes were 26–45%, 4–55% and 28–49%, respectively (Fig. 2, Table S2). However, the experimental values recorded after 24 h of CO_2_ exposure were indeed much less, being 9.9–15.6%, 25–29.4% and 5.6–24.17% w/w (total weight) in shell, fibre and soft peel ashes, respectively (Fig. 2, Table S2).

The crystalline phases observed in biomass ash included calcium oxide (CaO) and portlandite (Ca(OH)_2_). Calcium oxide and portlandite are the major elements responsible for reaction with CO_2_ under appropriate hydration condition. Hydration of CaO to portlandite (Ca(OH)_2_) is an extremely exothermic reaction (− 104 kJ/mol)^67^. On exposure to CO_2_, portlandite forms calcium carbonate (CaCO_3_) and this is similarly exothermic (− 32 kJ/mol)^68^, which liberates the formerly bound water^68,69^. Generally, > 10% w/w (total weight) of CaO in a material is associated with a CO_2_ uptake that leads to hardening by self-cementation via calcium carbonate formation.

**Figure 3:**
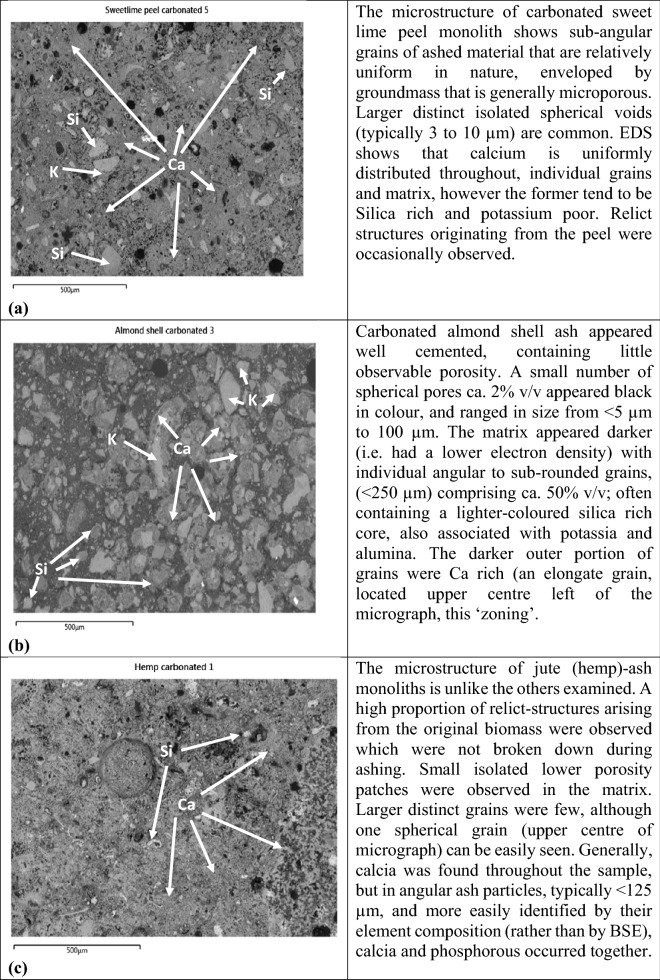
Back scattered micrographs and their descriptions (**a**) Sweet lime peel ash, (**b**) Almond shell ash and (**c**) Jute (hemp) ash.

Nam et al. (2012)^66^ suggest that as CO_2_ becomes imbibed, the reaction is predominantly controlled by the phase-boundary, whereas later on, it is controlled by the diffusion of CO_2_ through the surface carbonation reaction product. For fresh Portland cement, carbonation is essentially completed in three distinct phases involving 8 steps^66,70^. With respect to biomass ash, no pre-treatment was required, and the carbonation reaction involved: gaseous CO_2_ diffusing and dissolving into the film of moisture present on ash particles, followed by ionisation to HCO_3_^−^. As the pH of the moisture film (which now contains dissolved CaO) falls, as it is neutralised, CaCO_3_ is precipitated on the surface of ash particles, in pore space around and within the relict planty structures preserved in the ash. The formation of carbonate causes cementation of adjacent ash particles and the infilling of void space to produce a hardened monolithic product (see Fig. 3).

The observed maximum CO_2_ uptake was in ashes with the highest calcia content in the following order (low to high): jute (hemp), sweet lime, orange, banana and cassava peel, almond, walnut and cobnut shell, with a range from 10.43 to 29.45% w/w (total weight) (Fig. 2; Table S2). The calcia in these ashes was between 10.43 and 45.38% w/w (total weight), respectively (Table 3).

Carbon dioxide mineralisation of biomass ash was confirmed by X-Ray diffractometry by the presence of calcite. Rietveld refinement showed the relative occurrence of the major mineral phases in the biomass ashes and in their carbonated counterparts. As might be expected, the mineralogy of the ashes varied between the different biomass feedstock (Table 3). It should be noted that the mineralogy of the ashes is complex, and many amorphous phases are present. As such, the intensity of X-ray reflections is often lower than those obtained for mineral ashes from inorganic feedstock.

When exposed to CO_2,_ the ashes contained, for example, calcite and monohydrocalcite (observed for lime peel and nutshell). This clearly indicated that CO_2_ had been mineralised, and calcium carbonate was formed within the range: 14–67% w/w (total weight). For the sake of brevity, main phases taken from diffractograms of the ‘raw’ ash and its carbonated counterpart are presented for each category of biomass examined (Table 4).

**Table 4:** Example major calcium containing phases in carbonated biomass ashes (%w/w, total weight) as determined by X-ray diffractometry.

	Walnut shell	Jute (hemp)	Sweetlime peel
Lime (CaO)	–	–	–
Portlandite	33.07	–	–
Calcite	30.02	76.41	3.25
Monohydrocalcite	33.91	8.24	35.70
Hydroxylapatite	–	6.30	7.94

Data derived by Rietveld refinement; other phases detected included: periclase, quartz and feldspar.

The minor presence of portlandite and/or CaO was noted in some of the carbonated ashes indicating that complete carbonation had not been fully achieved, and that further exposure to CO_2_ was required. Some of the peel-derived ashes, such as banana and pomegranate were hygroscopic in nature and this could be attributed to the presence of sylvite (KCl); a phase that absorbs moisture from the air^71^. Portlandite development is also responsible for water sensitivity/expansion (and a relative loss of strength in carbonated monolithic specimens) when partially carbonated samples are immersed in water or exposed to the atmosphere^72^.

The literature has much information on the management of biomass ash and its effect on soil properties^73–75^, not least as a way of replenishing essential nutrients and for modifying soil structure/microstructure. The very nature of plants to accumulate metals shows there is a key relationship between soil chemistry/mineralogy and the ‘needs’ of individual plants. Where calcium is concerned, a plant’s ability to accumulate this divalent metal can result in ash that readily carbonates on exposure to CO_2_.

As mentioned, the uptake of CO_2_ in the biomass ashes was related to presence of CaO arising from the high ashing temperature of 900 ± 25 °C. The mineralisation of CO_2_ in the ashes was aided by their finely divided nature/high surface area, as noted by Castel et al. (2016)^76^ and Filho et al. (2009)^77^. Possan et al. (2017)^78^ reported that CO_2_ uptaken by, for example, mixed wood ash blended with coal is largely regulated by particle surface area. However, particle size is not always the limiting factor for the CO_2_ up taken, as is seen in our study. The findings of Nam et al. (2012)^66^ involving municipal solid waste ash showed the amount of CO_2_ sequestered increased as particle size decreased. This may well be valid for ashes with a similar chemistry, but where the amount of calcia varies in a feedstock (as seen in the present work) particle size, and in some cases, surface area may be secondary considerations.

### Ash only monoliths

The CO_2_ uptake in ‘ash-only’ monoliths (Table 5) showed that walnut shell, jute (hemp) and sweet lime peel reacted with the most CO_2_ in their respective categories. However, as noted, a small amount of residual CaO in banana peel ash caused water sensitivity-related micro-cracking as calcia hydrated to portlandite, with a consequent increase in the volume.

**Table 5:** Mechanical properties of biomass ash monoliths.

‘Ash-only’ monolith		Density (g/cm^3^)	CO_2_ uptake (g/kg)	Strength (MPa)	
				Carbonated	Uncarbonated
Shell	Cobnut shell	0.7	161.0	0.183	< 0.01
	Walnut shell	0.7	312.5	0.198	< 0.01
	Almond shell	0.7	342.0	0.507	< 0.01
	Peanut shell	0.4	156.2	0.169	< 0.01
Fibre	Jute (hemp)	0.5	325.0	0.147	< 0.01
	Flax	0.5	153.1	0.100	< 0.01
	Straw (barley)	0.5	78.0	0.161	< 0.01
	Hay	0.4	81.0	0.084	< 0.01
	Rice husk	0.2	− 9.95	0.028	< 0.01
	Coconut husk (coir)	0.4	20.5	– (Not cemented)	–
	Sugarcane husk	0.7	29.3	0.047	< 0.01
Soft peel	Sweet lime	0.6	283.0	0.313	< 0.01
	Banana	0.4	199.5	0.041	< 0.01
	Yam	0.4	136.8	0.022	< 0.01
	Cassava	0.5	147.0	0.256	< 0.01
	Potato	0.4	155.0	0.214	< 0.01
	Pomegranate	0.5	179.0	0.157	< 0.01
	Orange	0.6	273.0	0.299	< 0.01

Many of the biomass ashes studied have been shown to be very reactive to carbon dioxide gas forming calcium carbonate. The morphology of these carbonate products was examined using polished sections subject to backscattered electron microscopy. The spatial distribution of carbonate seen in the backscattered electron-micrographs suggests the nature of the individual biomasses may have an influence.

Three of the biomass residues, representing one type from each category studied, are given in Fig. 3a–c to illustrate the distinct morphology and carbonate distribution within the cemented biomass-ash monoliths. The observations made are summarised in each respective figure.

The ashing of biomass at 900 ± 25 °C produced primarily CaO as the main calcium-bearing phase. Other minor phases including quartz and feldspar were also noted, which were present in the raw biomass or formed during ashing.

**Figure 4:**
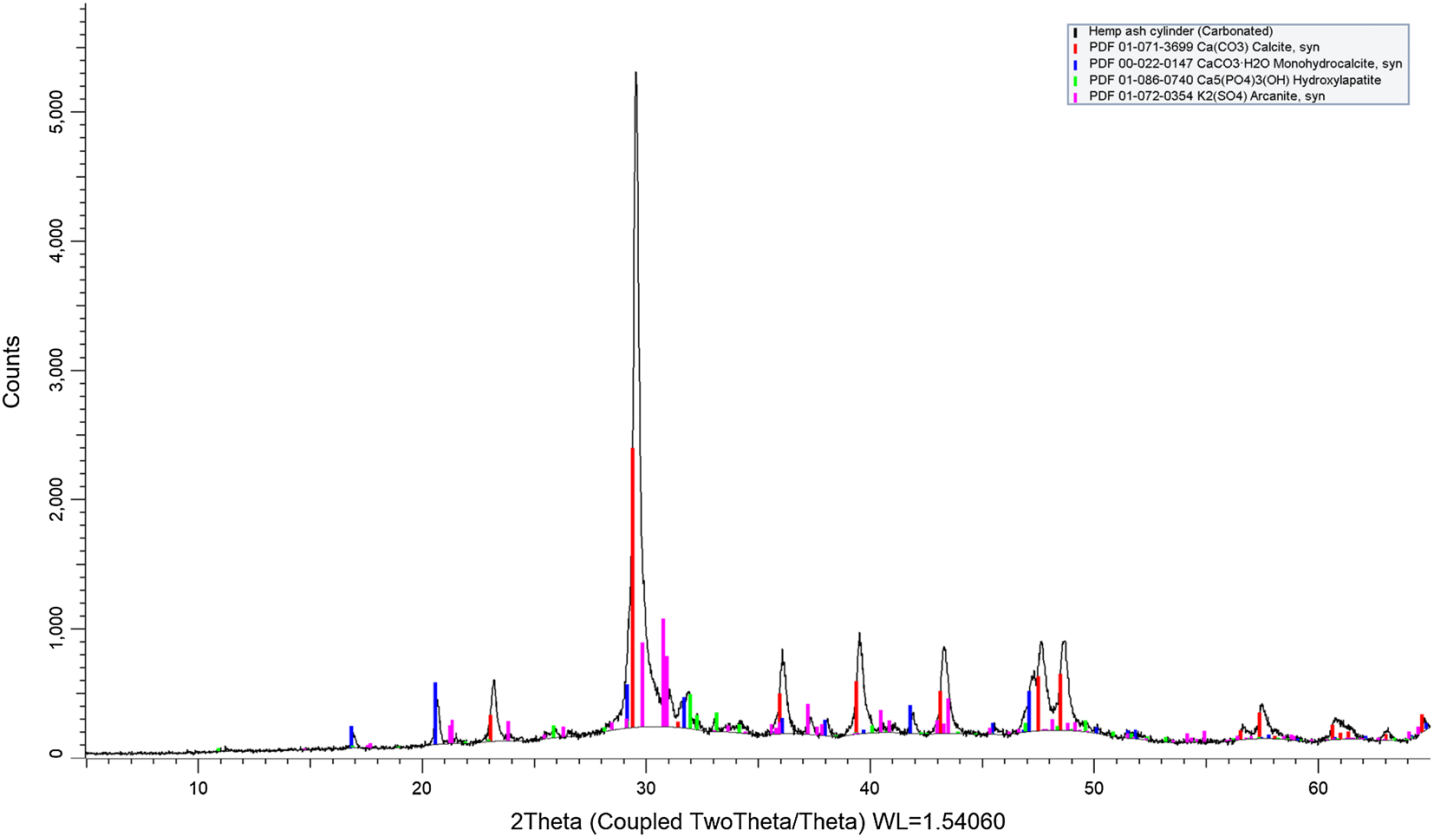
X-ray diffractogram showing polymorphs of carbonate in carbonated hemp ash cylinder/monolith.

It was also noted that monohydrocalcite was formed in combination with calcite during carbonation. Monohydrocalcite is a metastable phase which can transform to calcite or aragonite under the right environmental conditions. However, small amounts of phosphate can significantly inhibit transformation, and may explain why some of the samples contained both hydroxylapatite and monohydrocalcite^79^. An example diffractogram showing these phases is given in Fig. 4.

Overall, the biomass ashes investigated were CO_2_ reactive, and displayed a self-cementing capacity resulting in a hardened mineralised material. Interestingly, the variations in the microstructure of the mineralised products readily confirms carbonate cementation upon reaction with CO_2_ gas. Furthermore, the possibility of achieving carbon balance/neutrality in the encapsulated biomass residues with sequestered CO_2_ is established.

### Raw biomass and ash monoliths

The embodied carbon calculated in monoliths produced from raw biomass combined with CO_2_-reactive biomass ashes was 10–20% w/w (total weight). For cement-bound composites the amount measured was 10% w/w (total weight), clearly indicating (that despite containing the cement), the monoliths were carbon negative (Table 6). The mechanical properties of the ash + raw biomass-only monoliths without cement were inferior to those containing cement.

**Table 6:** Mechanical properties of a few biomass ash containing raw biomass monoliths (Tripathi and Hills et al. 2020) ^54^.

Combinations	Density of valorised raw biomass and ash products (carbonated) (g/cm^3^)	Strength of valorised raw biomass and ash products (carbonated) (MPa)	CO_2_ in valorised raw biomass and ash products (1-week carbonation) (%)
Orange peel + cement (20%) + sand (10%)	400	0.230	45.1
Orange peel + Poplar shavings ash, cement and sand (10% each)	350	0.160	43.6
Hazelnut shell + cement (20%) + sand (10%)	880	0.450	28.6
Hazelnut shell + Hazelnut shell ash, cement and sand (10% each)	740	0.280	26.4

When the CO_2_-reactive biomass ashes were used as a partial replacement for cement (see Table 6), it is possible to obtain respectable strengths and a reduced carbon footprint for the products formed. This particular part of our wider study will be published separately.

## Strength of carbonated monolith samples

The carbonated ‘ash only’ monolithic cylinders were examined for their unconfined compressive strength and density (Table 5). The strength achieved was greatest for almond shell, straw and sweet lime in their respective categories. As compared to uncarbonated monolithic samples, all the carbonated biomass ashes were stronger. The monoliths made from rice and sugarcane husks, and yam peel recorded the lowest strengths, and this appears to be related to raised silica content (15–65% w/w, total weight). Interestingly, coconut husk ash poorly self-cemented (despite having a modest silica content) and this was attributed to the formation of sylvite (KCl) in the hardened product.

**Figure 5:**
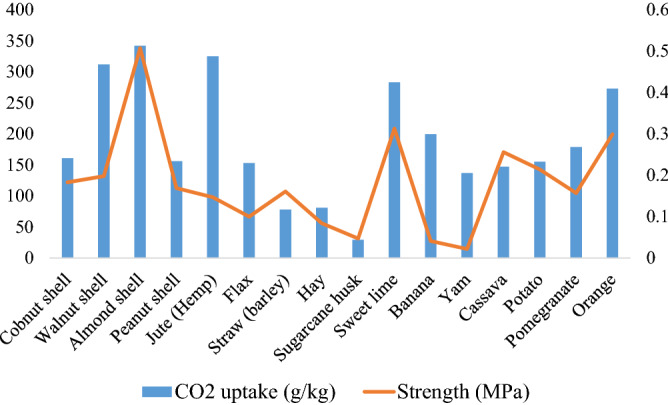
Relationship between strength and CO_2_ uptake in monoliths from biomass residues.

In most of the cases, the uptake of CO_2_ in ashes corresponded to the strength recorded for the carbonate cemented monoliths (i.e. higher CO_2_ uptake = higher strength). However, in some cases, e.g., for banana peel a high CO_2_ uptake resulted in a low recorded strength; here being attributed to incomplete carbonation leading to mild expansion and microcracking (Fig. 5).

As a means of comparing how the strengths of the small monolithic specimens made from carbonated biomass ash with commercially manufactured carbonated products reference is made to the European standard for lightweight aggregates^80^. It was found that the strength of all the carbonated ash-monoliths examined exceeded the strength criteria given in this standard, being an average of 0.1 MPa. This strength is also that required for ‘end of waste’ approval for UK commercially available manufactured carbonated aggregate-products, made from CO_2_ reactive inorganic wastes^81^.

## Implications

Some of the nutshell, fibre and soft peel ashes have displayed potential to combine with ca. 25% of their own weight of CO_2_. However, most of the fibre-ashes captured much less CO_2_. Nevertheless, the amount of captured CO_2_ is the direct offset of the CO_2_ emitted during ashing.

The development of strength by self-cementation by carbonate formation may not necessarily be sufficient for biomass ash raw-biomass composites to be employed as building materials. Under these circumstances a more ‘potent’ carbonate-able binder may be used, and Portland cement is one such binder.

As shown in this work, when Portland cement is used as a carbonate-able binder, replacement by biomass ash can be used to increase the embodied carbon in the product whilst maintaining desirable strength characteristics. It is worthy of note from our earlier work^54^, Ca-rich biomass ash from woody feedstock could be used to partially replace Portland cement in a hydraulic system without loss of product strength, providing flexibility in approach.

The strengths observed for biomass ash monoliths and our reference cement-bound samples ranged between 160 and 450 kPa. Considering that the composites were raw biomass at 70% w/w (total weight), these strengths were enough for the samples to be robustly handled. There is also a considerable amount of embodied carbon in these composite products, ranging between 26 and 45% w/w (total weight).

An emission factor for CO_2_ emissions from burning of crop residues has been calculated as 1585 g/kg by Akagi et al. (2011)^82^. However, under the laboratory conditions used in this work, we calculated the emissions from burning of crop residues to be 47–66% w/w (total weight) CO_2_, which is lower than that reported by these authors using a CO_2_ equivalent calculation.

The direct offset of CO_2_ emissions after carbonation of ashes (i.e. what was mineralised/in the raw biomass) could be calculated based on the CO_2_ mineralised the products.

The indirect offset, when Portland cement is used (as a carbonate-able binder), can be calculated through the reduction in use of cement by partial replacement by biomass ash. Further potential benefits include release of land space currently used for dumping biomass residues.

**Figure 6:**
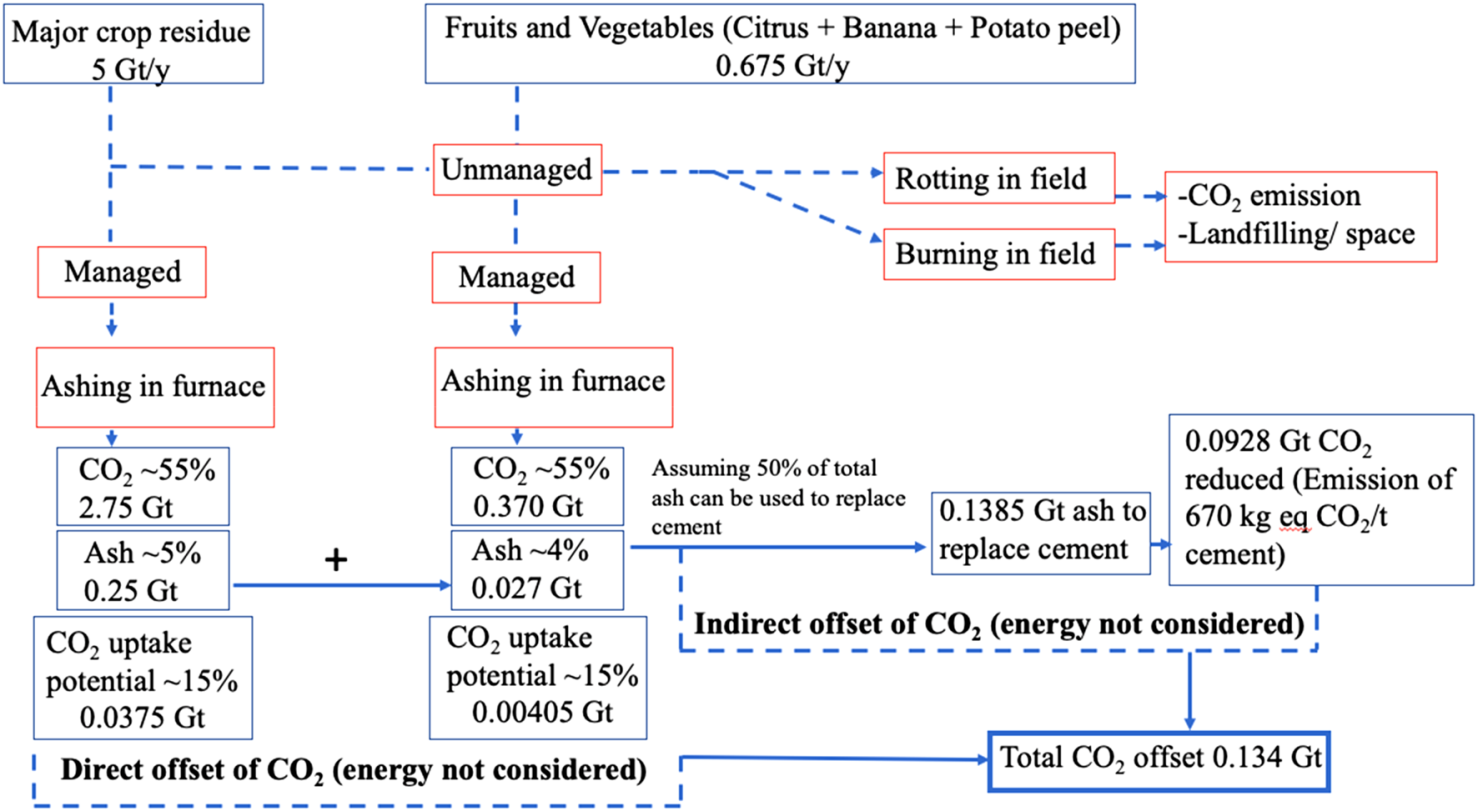
A conceptual diagram for CO_2_ emission offset envisaged from CCUS option for biomass waste valorisation.

A simplified conceptual diagram shown in Fig. 6 delineates the offset options for CO_2_ emission by using our low carbon CCUS approach for the valorisation of selected biomass waste. This diagram, however, does not consider the energy involved in burning biomass, as that is the part of a separate study. Nevertheless, direct and indirect offsets calculated for selected biomass residues amount to 134 Mt of CO_2_/year.

## Conclusion

The ‘proof of concept’ established through this study shows that the residual ash from the burning of certain agricultural biomass waste contains enough calcium oxide (× 100 g/kg), to enable carbonate-hardened monolithic products to be manufactured on exposure to CO_2_ gas. A negative relationship between calcia content and ash yield was identified. When fully carbonated, these small monolithic products similar in size to manufactured carbonated aggregate are resistant to water and have acceptable strength, as specified in the European standard for light-weight aggregates, BSEN 13055:2016.

These findings suggest an alternative ‘low carbon’ route for biomass waste utilisation is potentially available, which can sequester significant (× 100 Mt) amounts of CO_2_ in products with value.

We conclude from this study that there are a number of significant potential benefits from utilising biomass waste ash that has been mineralised:

- significant amounts of CO_2_ can be permanently stored, being up to 29.5% w/w, total weight
- processing can be carried under ambient conditions opening up the possibility of using point-source emissions at low cost
- ashes readily self-cement and the products have MPa strength, and appear environmentally stable
- biomass ash wastes normally disposed to landfill under waste management regulations have a route for valorisation via ‘end of waste’
- the combining of solid and gaseous wastes in products is a circular economic activity with significant potential sustainability gains
- The combined direct and indirect CO_2_ offset was calculated as 134 Mt/year for the selected biomass residues (incl. crop, fruit and vegetable waste); this amount is equivalent to, e.g., nearly 40% of the UK’s GHG emissions predicted for 2019.

## Supplementary information

Supplementary information
