# Supplementary material for: Valorisation of agricultural biomass-ash with CO2
Source: Sci Rep. 2020 Aug 14;10:13801. doi: 10.1038/s41598-020-70504-1 (PMC7429819; doi:10.1038/s41598-020-70504-1)
Supplement: Supplementary file 1 — Supplementary information [file 41598_2020_70504_MOESM1_ESM.docx]

**Valorisation of agricultural biomass-ash with CO_2_**

**Colin D Hills*, Nimisha Tripathi *, Raj S Singh^1^, Paula J Carey and Florence Lowry**

Indo:UK Centre for Environment Research and Innovation, University of Greenwich, Chatham Maritime, UK

^1^Indo:UK Centre for Environment Research and Innovation, Central Institute of Mining and Fuel Research, Dhanbad, India

*Email : [c.d.hills@gre.ac.uk](mailto:c.d.hills@gre.ac.uk); [n.tripathi@gre.ac.uk](mailto:n.tripathi@gre.ac.uk)

**Supplementary material**

**Table S1:** **Cumulative CO_2_ uptake (w/w, total weight) in agricultural biomass ashes after 4 carbonation cycles**

| Type |  | CO_2_ uptake (g/kg)^#^ | | | |
| --- | --- | --- | --- | --- | --- |
|  |  | 1 hr | 1 hr | 1 hr | 24 hrs |
| **Shell** | Cobnut shell* | 62.3 | 64.4 | 82.9 | 113.7 |
|  | Walnut shell | 35.4 | 64.0 | 64.3 | 141.0 |
|  | Almond shell* | 69.7 | 82.2 | 84.1 | 155.6 |
|  | Peanut shell | 32.0 | 65.2 | 72.0 | 99.3 |
| **Fibre** | Jute (hemp)* | 188.0 | 216.6 | 218.5 | 294.5 |
|  | Flax | 74.1 | 82.5 | 88.6 | 115.9 |
|  | Straw (barley) | 17.0 | 36.3 | 37.0 | 48.6 |
|  | Hay | 2.4 | 3.7 | 5.3 | 9.3 |
|  | Rice husk | 2.4 | 2.5 | 2.9 | 4.4 |
|  | Coconut husk (coir) | 5.4 | 5.4 | 8.2 | 12.9 |
|  | Sugarcane husk | 2.7 | 3.8 | 4.3 | 9.8 |
| **Soft peel** | Sweet lime | 41.6 | 55.6 | 85.3 | 241.7 |
|  | Banana | 34.6 | 96.8 | 101.7 | 110.9 |
|  | Yam | 32.7 | 42.7 | 51.0 | 56.0 |
|  | Cassava | 86.7 | 99.9 | 103.1 | 127.2 |
|  | Potato | 58.0 | 58 | 51.9 | 75.0 |
|  | Pomegranate | 87.4 | 87.4 | 101.0 | 135.8 |
|  | Orange* | 67.3 | 104.9 | 149.6 | 150.0 |

*****ref materials

^#^ To convert g/kg to %, divide by 10

**Table S2: Theoretical and experimental CO_2_ uptake in biomass ashes**

| Biomass residue | | CO_2_ emitted during ashing of biomass  (% w/w, total weight) | Predicted (Steinour) CO_2_ uptake (based on ash composition)  (% w/w, total weight) | CO_2_ in ash (after 24 hrs of CO_2_ exposure) (% w/w, total weight) | Difference between predicted and experimental CO_2_ uptake (% w/w, total weight) |
| --- | --- | --- | --- | --- | --- |
| **Shell** | Cobnut shell | 64.0 | 33.9 | 13.6 | 20.3 |
|  | Walnut shell | 54.2 | 48.9 | 19.8 | 29.1 |
|  | Almond shell | 66.0 | 34.9 | 18.2 | 16.7 |
|  | Peanut shell | 59.0 | 26.4 | 7.2 | 19.2 |
| **Fibre** | Jute (hemp) | 58.8 | 55.0 | 29.4 | 25.6 |
|  | Flax | 60.4 | 28.4 | 13.9 | 14.5 |
|  | Straw (barley) | 52.7 | 30.5 | 8.8 | 21.7 |
|  | Hay | 53.8 | 24.1 | 1.3 | 22.8 |
|  | Rice husk | 49.0 | 4.4 | 0.5 | 3.9 |
|  | Coconut husk (coir) | 58.2 | 19.2 | 1.6 | 17.6 |
|  | Sugarcane husk | 59.0 | 14.5 | 1.2 | 13.3 |
| **Soft peel** | Sweet lime | 49.2 | 47.9 | 30.0 | 17.9 |
|  | Banana | 47.0 | 35.9 | 24.0 | 11.9 |
|  | Yam | 49.7 | 49.6 | 8.43 | 41.17 |
|  | Cassava | 60.7 | 25.3 | 15.7 | 9.6 |
|  | Potato | 51.3 | 27.9 | 6.9 | 21 |
|  | Pomegranate | 51.5 | 31.4 | 14.9 | 16.5 |
|  | Orange | 49.2 | 47.9 | 27.0 | 20.9 |
